# Ultrasound-induced structural remodeling of plant proteins for high-internal-phase Pickering emulsion stabilization: A review

**DOI:** 10.1016/j.fochx.2026.104663

**Published:** 2026-10-20

**Authors:** Uyory Choe

**Affiliations:** Department of Food and Nutrition, College of Biomedical and Health Science, Konkuk University, Chungju 27478, Republic of Korea

**Keywords:** High-intensity ultrasound, High-internal-phase Pickering emulsion, Plant protein

## Abstract

High-intensity ultrasound has been used in the preparation of plant-protein-based high-internal-phase Pickering emulsions (HIPPEs) by improving protein dispersion, modifying particle properties, and facilitating droplet breakup and interfacial adsorption. However, its effects depend on when ultrasound is applied during emulsion preparation. Ultrasound may be used as protein pre-sonication before oil addition, applied during emulsification, or introduced after emulsification to reorganize an already formed droplet network. These routes can produce distinct structural and functional outcomes. Ultrasound effects are also influenced by acoustic power, treatment time, protein concentration, oil fraction, sample volume, and temperature control. In this review, ultrasound-assisted HIPPE studies are compared according to these three treatment stages. Additionally, the combined use of ultrasound and other protein modification methods is discussed. Changes in protein structure and droplet characteristics are linked to interfacial adsorption, droplet crowding, and continuous-phase structuring. Protein-to-oil ratio, oil fraction, process scale-up, and validation in food applications are also discussed.

## Highlights

• Ultrasound timing governs plant-protein remodeling and HIPPE stabilization.
• Pre-, direct-, and post-emulsification routes produce distinct outcomes.
• Protein distribution between interface and continuous phase shapes HIPPE stability.
• Optimal ultrasound conditions depend on protein state and formulation.
• Scale-up requires controlled acoustic exposure and product validation.

## Graphical abstract

**Figure:**
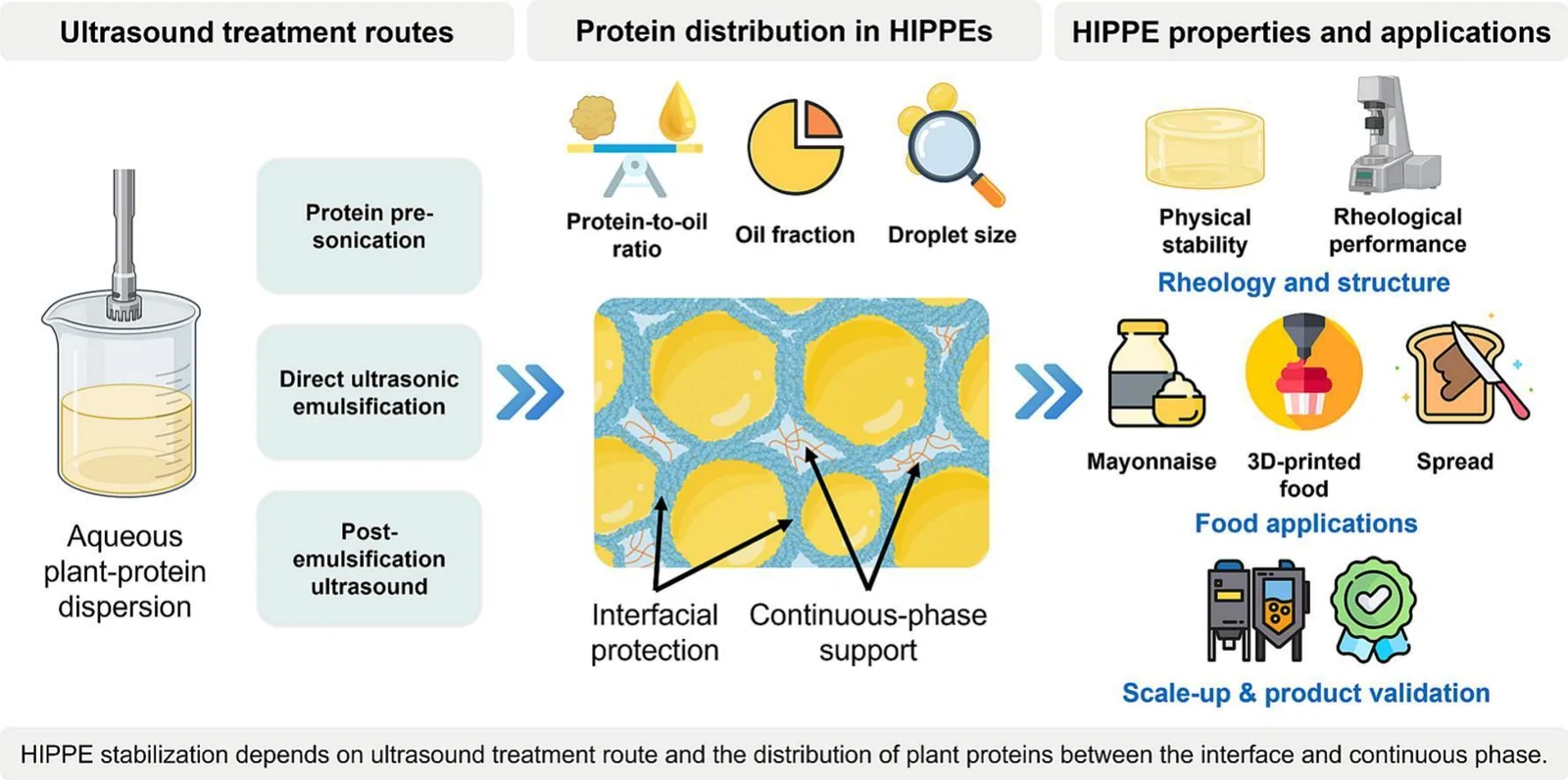

## 1 Introduction

Emulsions are dispersions of two immiscible liquids in which one phase is distributed as droplets within the other. In food systems, oil-in-water emulsions are widely used to structure products such as sauces, dressings, beverages, and spreads, although their physical stability can be compromised by flocculation, coalescence, creaming, and oil separation during processing and storage. Pickering emulsions use colloidal particles that adsorb at the oil–water interface and form a protective interfacial layer, providing an alternative means of stabilizing dispersed droplets. High-internal-phase Pickering emulsions (HIPPEs) extend this concept to systems containing a dispersed oil phase near or above the conventional close-packing reference of 0.74. As the oil fraction increases, droplets become crowded and deform, while the aqueous phase is confined to thin films and channels. The resulting materials can exhibit yield stress, shear thinning, and solid-like viscoelasticity and have been investigated for fat replacement, spreads, printable foods, and delivery of lipophilic compounds (He & Lu, 2024; Ji & Luo, 2023; Tian et al., 2024).

Among food-grade stabilizers used for HIPPE formation, plant proteins can contribute to multiple stabilization processes through their amphiphilic nature and ability to form diverse colloidal assemblies. These assemblies, including colloidal particles, microgels, heat-set aggregates, and fibrillar structures, may adsorb at the oil–water interface and resist coalescence, whereas unadsorbed material can increase continuous-phase viscosity, bind water, bridge adjacent droplets, or form a separate network (Aryanti et al., 2025b; He & Lu, 2024; Ji & Luo, 2023; Liang et al., 2025; Zhang, Li, Zhou, et al., 2023). However, it remains unclear how ultrasound changes the distribution and function of proteins between the interface and the continuous phase under different treatment routes and formulation conditions.

Plant-protein preparations contain proteins in different structural and aggregation states. These proteins exist as soluble molecules, dispersed colloidal particles, or larger insoluble aggregates, with their distribution influenced by processing history. Ultrasound can modify these protein populations through aggregate fragmentation, improved hydration, exposure of hydrophobic or charged regions, and increased molecular flexibility. Depending on the treatment intensity and duration, ultrasound may also promote reassociation or oxidation. As a result, ultrasound can reshape the distribution and structural properties of these protein populations, influencing both interfacial stabilization and continuous-phase structuring in HIPPEs (Das et al., 2022; Gharibzahedi & Smith, 2020; Jadhav et al., 2024; O'Sullivan et al., 2016). This heterogeneity complicates the interpretation of ultrasound-induced effects because changes in HIPPE stability could reflect alterations in several protein populations and their respective roles at the interface and in the continuous phase.

When ultrasound is applied during HIPPE formation, protein restructuring occurs together with droplet breakup and the formation of new interfaces. Smaller droplets increase the total oil–water interfacial area and require more protein to cover the newly generated surface. Ultrasound also enhances mixing and promotes the transport of proteins toward the interface, although recoalescence may occur when new surface is generated faster than proteins can adsorb. Protein concentration and continuous-phase viscosity can further affect cavitation, liquid circulation, and droplet disruption. These coupled effects on droplet size, interfacial adsorption, droplet crowding, and continuous-phase structuring make it difficult to identify the dominant factor responsible for improved HIPPE stability (Sui et al., 2017).

Ultrasound effects do not increase monotonically with power or treatment time. Studies involving ultrasound treatment of faba bean, pea, and chickpea proteins have shown that intermediate treatment intensities or durations can improve colloidal and interfacial properties, whereas excessive treatment can promote reaggregation or reduce functionality (Cui et al., 2025; Lee et al., 2025). The optimal treatment conditions, in turn, vary with protein source, initial aggregation state, concentration, sample volume, and ultrasound configuration.

Ultrasound conditions explain only part of the variation among reported HIPPE systems because formulation also affects the outcome (J. Zhang, Zhao, Li, et al., 2023; Badar, Wang, Sun, et al., 2024). The same aqueous protein concentration can correspond to different protein-to-oil ratios as oil fraction changes, while differences in droplet size alter the total interfacial area that must be stabilized (Lee et al., 2025; J. Zhang, Zhao, Li, et al., 2023). In addition, adsorbed and unadsorbed protein fractions are rarely quantified together, limiting assessment of the relative contributions of interfacial adsorption and continuous-phase structuring (Li, Liu, et al., 2025; Wen, Zhao, Zhang, et al., 2024). Although bulk viscosity, G′, water-holding capacity, and visual stability provide useful information on overall emulsion behavior, they do not distinguish the contributions of droplet crowding, bridging, continuous-phase structuring, and interfacial properties (Badar, Wang, Chen, et al., 2024; L. Zhu et al., 2025). Thus, identifying where the protein is located and how it contributes to HIPPE structure is essential for clearer interpretation of the stabilization mechanism.

Previous reviews have summarized plant-protein HIPPEs according to protein source, particle preparation, modification methods, rheological properties, and food applications (Aryanti et al., 2025b; He & Lu, 2024; Ji & Luo, 2023; Liang et al., 2025; Tian et al., 2024; M. Zhang et al., 2023), while broader ultrasound reviews have mainly focused on protein modification and emulsification (Das et al., 2022; Gharibzahedi & Smith, 2020; Jadhav et al., 2024; O'Sullivan et al., 2016). However, limited attention has been given to how the stage of ultrasound treatment affects protein restructuring, interfacial behavior, and HIPPE stabilization. The present review compares three ultrasound treatment routes, including protein pre-sonication, direct ultrasonic emulsification, and post-emulsification ultrasound, and examines how the timing of ultrasound application influences protein modification and HIPPE stabilization. It also considers how treatment and formulation conditions influence the reported outcomes, together with process optimization, scale-up, and validation in food applications.

## 2 Ultrasound processing routes at different treatment stages

In protein pre-sonication, the aqueous protein dispersion is treated with ultrasound before oil is added. This approach allows ultrasound-induced changes in protein properties to be examined before emulsification, without the simultaneous effects of droplet breakup. This means that changes in particle size, solubility, morphology, and surface properties can be directly evaluated after sonication. The type and extent of these changes vary with ultrasound power and treatment time. For example, studies using heat-treated faba bean and pea proteins (Lee et al., 2025), chickpea protein (Cui et al., 2025), and acid-treated soybean lipophilic protein (Y. Sun et al., 2022) have shown that moderate treatment can improve protein dispersion, while excessive treatment may lead to reaggregation. A limitation of this approach, however, is that the protein state may continue to change during cooling or storage before oil addition, so properties measured immediately after sonication may not fully represent those present at emulsification.

Unlike protein pre-sonication, direct ultrasonic emulsification applies ultrasound after the aqueous protein dispersion is combined with oil. In this route, proteins, oil droplets, and the newly forming interface are exposed to cavitation simultaneously. This route combines protein modification and droplet breakup within a single process. However, the simultaneous occurrence of these processes makes it difficult to determine whether improved emulsion properties result from changes in protein structure, droplet size, or both. This complexity is illustrated by studies of soy protein–lecithin emulsions and protein–polysaccharide systems, where droplet breakup, protein adsorption, and continuous-phase structuring can occur simultaneously (Sui et al., 2017; Sun, Li, et al., 2025; Wang et al., 2022; Zhang, Li, Sun, et al., 2023). To distinguish these effects, direct ultrasonic emulsification should be compared with a conventionally emulsified control without ultrasound as well as a temperature-matched control that accounts for heating during sonication.

A third route is post-emulsification ultrasound, in which ultrasound is applied after an initial emulsion has formed. Unlike direct ultrasonic emulsification, this treatment acts on existing droplets and on interfacial or continuous-phase structures that have already developed. This route can be effective when the initial emulsion contains coarse or flocculated droplets and enough protein remains in the continuous phase to adsorb onto newly generated interfaces. Under these conditions, post-emulsification ultrasound can reduce flocculation and further decrease droplet size. In contrast, the method can be problematic when the interfacial layer is already densely packed or when little protein remains available in the continuous phase, because protein may not adsorb rapidly enough to cover newly generated interfaces. Excessive treatment can also disrupt an established interfacial layer. These outcomes depend on both the initial state of the emulsion and the ultrasound treatment conditions. Thus, the pre-emulsion composition, initial droplet size, delivered acoustic energy, and post-treatment temperature should be clearly reported when comparing studies (Diao et al., 2024; C. Liu et al., 2025; Wang et al., 2022).

In addition to these three treatment routes, ultrasound can be combined with other processing or modification strategies, including pH shifting, heating, polysaccharide or polyphenol incorporation, glycation, and enzymatic treatment. In these hybrid treatments, the order of processing is important because ultrasound can act on proteins before, during, or after the secondary modification, resulting in different protein structures and functional properties (Aryanti et al., 2025a; Chen et al., 2023; X. Chen et al., 2022; Cui et al., 2026; Fazelioskouei et al., 2023; Huang et al., 2024; Jiang et al., 2022; S. Li et al., 2024; Li et al., 2025; B. Liu et al., 2026; X.-Y. Liu et al., 2024; Ma et al., 2025; Nimaming et al., 2024; Y. Song et al., 2025; X. Sun et al., 2023; Y. Sun et al., 2024; Sun, Dong, et al., 2025; N. Wang et al., 2023; Wang et al., 2022; Wang et al., 2022; Wang et al., 2022; Xu et al., 2024; Ye et al., 2025; Yu et al., 2025; J. Zhang et al., 2022; J. Zhang, Zhao, Liu, et al., 2023; R. Zhang et al., 2025; L. Zhu et al., 2025). Table 1 summarizes the three ultrasound treatment routes, their main structural outcomes, key advantages, and interpretive limitations.

**Table 1:** Comparison of ultrasound processing routes used to modify plant proteins and form Pickering emulsions or HIPPEs.

Processing route	When ultrasound is applied	Primary acoustic target	Main structural outcome	Best suited for/key advantage	Main interpretive limitation	References
Protein pre-sonication	Aqueous protein dispersion is treated before oil addition.	Native aggregates and protein assemblies.	Fragmentation, hydration, partial unfolding, dissociation, and/or post-treatment reassembly.	Useful when the effects of ultrasound on the protein stabilizer need to be examined separately from droplet formation; permits independent particle characterization.	Particle state may change during cooling, equilibration, pH adjustment, ingredient addition, or later homogenization.	Lee et al., 2025; Cui et al., 2025; Y. Sun et al., 2022
Direct ultrasonic emulsification	Protein dispersion and oil are sonicated together.	Coarse droplets, newly forming interfaces, and protein structures.	Simultaneous droplet breakup, protein restructuring, adsorption, and recoalescence competition.	Suitable for combining protein modification and emulsification in a single processing step.	Improved stability does not prove Pickering stabilization; molecular films and bulk thickening may dominate.	Sui et al., 2017; T. Wang et al., 2022; L. Zhang et al., 2023; J. Sun et al., 2025
Post-emulsification ultrasound	Ultrasound is applied after a coarse or preformed emulsion has been produced.	Droplet clusters, existing interfaces, and unadsorbed stabilizers.	Secondary droplet breakup, deflocculation, redistribution of proteins, or disruption/reorganization of established layers.	Useful for refining coarse or flocculated preformed emulsions when protein remains available in the continuous phase to cover newly generated interfaces.	May damage a previously formed particle layer or generate new uncovered interface.	Diao et al., 2024; C. Liu et al., 2025; T. Wang et al., 2022

## 3 Changes in plant-protein state after ultrasound treatment

Plant proteins used in HIPPE systems can exist as soluble molecules, dispersed colloidal particles, or larger insoluble aggregates, and these populations may respond differently to cavitation. Bubble collapse and microstreaming can disrupt weak associations and improve hydration, while newly exposed interaction sites may promote reassociation during or after treatment. For this reason, the structural and aggregation states of proteins should be characterized at the time of emulsification because additional changes may occur after sonication.

### 3.1 Aggregate breakdown and surface property changes

Acoustic cavitation generates microstreaming, shear forces, pressure fluctuations, and repeated particle collisions. These physical effects can disrupt noncovalent interactions within large protein aggregates, including hydrogen bonding, hydrophobic interactions, and electrostatic interactions. As a result, ultrasound treatment can reduce turbidity and sedimentation while improving protein solubility (Das et al., 2022; Gharibzahedi & Smith, 2020; Guo et al., 2025; Jadhav et al., 2024; Kim & Shin, 2022; O'Sullivan et al., 2016; Xu et al., 2024). The extent of these changes depends on the protein's initial aggregation state and the ultrasound treatment conditions.

These changes in aggregate size should be interpreted carefully. A reduction in measured particle size after ultrasound does not necessarily indicate complete dissociation of large aggregates into individual protein molecules. Ultrasound can fragment large protein aggregates into smaller assemblies without fully dissociating them into individual protein molecules. In some cases, however, aggregates can dissociate into individual proteins or small oligomers. Both changes can increase the measured solubility after centrifugation, so solubility measurements cannot fully distinguish between aggregate fragmentation and more extensive dissociation. Thus, particle-size distribution and morphology should be examined to determine whether ultrasound causes aggregate breakdown or more extensive protein dissociation.

Even when ultrasound promotes aggregate breakdown, the extent of this effect does not necessarily increase with treatment intensity. At moderate treatment levels, ultrasound improved the dispersion of black soybean protein, oxidized soybean aggregates, and insoluble potato protein, while stronger treatment of oxidized soybean aggregates promoted reaggregation (Kim & Shin, 2022; Y. Wang et al., 2022; Zhao et al., 2022). A similar response was observed in chickpea protein. At 20 kHz for 10 min, increasing ultrasound power up to 450 W progressively reduced particle size, whereas treatment at 600 W increased particle size and reduced emulsion performance (Cui et al., 2025). These findings show that terms such as “moderate” or “intermediate” treatment should be defined using the reported power, treatment time, sample conditions, and temperature control.

Changes in aggregation state can also alter surface properties of plant proteins by exposing hydrophobic, charged, and sulfhydryl-containing regions. Moderate exposure of hydrophobic regions can improve interactions with the oil phase while maintaining sufficient hydration in the aqueous phase. Excessive exposure may increase protein–protein interactions and promote reaggregation (Cui et al., 2025; Lee et al., 2025; Y. Wang et al., 2022). Changes in sulfhydryl accessibility can influence network formation through thiol–disulfide exchange. The presence of other components during ultrasound treatment can further modify these surface properties. For example, studies using soybean protein modified with L-histidine and soy protein–pectin complexes showed that the resulting surface properties depended on both the protein structure and the co-component present during treatment (N. Wang et al., 2022; Xu et al., 2024; J. Yang et al., 2023).

Changes in surface properties may be accompanied by changes in protein secondary structure. Secondary-structure analysis can provide information on these conformational changes, but the result depends on the protein fractions included in the analysis. When soluble proteins, dispersed colloids, and sedimentable aggregates are analyzed together, the measured spectrum represents an average of these fractions and may mask larger changes within individual fractions. In addition, secondary-structure measurements do not identify which protein structures are present at the oil–water interface. In this regard, secondary-structure data should be interpreted together with particle fractionation, morphology, and interfacial measurements.

Together, changes in aggregation state, surface properties, and secondary structure can influence how plant proteins contribute to HIPPE stabilization. Smaller and more deformable particles can adsorb efficiently at curved oil–water interfaces, while larger aggregates may promote droplet bridging or strengthen the continuous phase. Thus, minimizing the mean particle size should not be treated as the primary goal of ultrasound treatment. Instead, ultrasound conditions should be selected to produce particles with suitable size, surface properties, and flexibility for interfacial adsorption and continuous-phase structuring (Han et al., 2024; Hei et al., 2024; Ji et al., 2025; C. Liu et al., 2025; Shen et al., 2024; Wei et al., 2025).

### 3.2 Reassociation and stability after ultrasound treatment

After sonication, newly exposed protein regions can continue to interact and promote reassociation. These changes may occur during cooling or storage before emulsification and can alter the size and aggregation state of the protein particles. As a result, the particle size measured immediately after sonication may not reflect the protein state at the time of emulsification.

The extent of protein reassociation following sonication is an important factor influencing colloidal and interfacial properties. Limited reassociation may preserve particle cohesion while maintaining sufficient mobility and deformability for interfacial adsorption. In contrast, extensive reassociation can increase aggregate size and compromise interfacial functionality. For example, in heat-treated faba bean and pea protein dispersions, 4% (*w*/*v*) protein was heated at 90 °C for 2 h and then sonicated at 20 kHz and 120 W for 5, 10, or 20 min. Treatments for 5–10 min produced favorable colloidal and interfacial properties, while 20 min caused partial reaggregation and reduced interfacial efficiency (Lee et al., 2025). Similar observations have been reported for ultrasound-induced quinoa protein particles, supporting the importance of controlling reaggregation to maintain favorable colloidal and interfacial properties (Qin, Luo, & Peng, 2018; Qin, Luo, Peng, Lu, et al., 2018; X. Zhang et al., 2021).

When reassociation becomes excessive, the particle size distribution can become broader, sedimentation can increase, and interfacial coverage may become less uniform. This shift from aggregate fragmentation to reaggregation was observed for chickpea protein at 20 mg/mL in a 100 mL batch (Cui et al., 2025). The samples were treated at 20 kHz for an effective 10 min at powers ranging from 150 to 600 W under ice-water cooling. Particle size decreased as the power increased up to 450 W, which produced the smallest particles and the highest Pickering-emulsion stability. At 600 W, particle size increased again because of reaggregation, accompanied by reduced emulsion stability. These results demonstrate the importance of identifying ultrasound conditions that promote aggregate fragmentation without inducing excessive reaggregation.

When ultrasound is combined with other processing or modification strategies, treatment order becomes an important variable because the protein state entering sonication can differ depending on prior treatment. Ultrasound combined with pH shifting has improved pea-protein solubility and emulsifying properties, with the resulting protein state influencing protein adsorption at the oil–water interface during emulsification (J. Zhang et al., 2022; J. Zhang, Zhao, Liu, et al., 2023). In systems containing added biopolymers or polyphenols, changes in particle association, interfacial adsorption, and continuous-phase structuring can further complicate attribution of the observed effects to ultrasound. These responses depend on component ratios, pH, and treatment sequence (Aryanti et al., 2025a; Chen, Pan, et al., 2023; X. Chen et al., 2022; Cui et al., 2026; Fazelioskouei et al., 2023; Huang et al., 2024; Igartúa et al., 2024; Jiang et al., 2022; Li, Wang, et al., 2025; B. Liu et al., 2026; X.-Y. Liu et al., 2024; Lv et al., 2025; Ma et al., 2025; Nimaming et al., 2024; Rawal et al., 2025; Y. Song et al., 2025; X. Sun et al., 2023; Y. Sun et al., 2024; Z. Sun et al., 2025; N. Wang et al., 2023; S. Wang et al., 2022; W. Wang et al., 2022; Ye et al., 2025; Yu et al., 2025; R. Zhang et al., 2025). To interpret such hybrid systems, the components present at the oil–water interface and in the continuous phase should be identified separately.

Fig. 1 summarizes ultrasound-induced changes in plant-protein state, including aggregate fragmentation, dissociation, and reassociation, and illustrates the distribution of the resulting protein populations during HIPPE formation. The resulting proteins and particles may adsorb at the oil–water interface or remain in the continuous phase, where they can contribute to network formation and HIPPE stability.

**Fig. 1:**
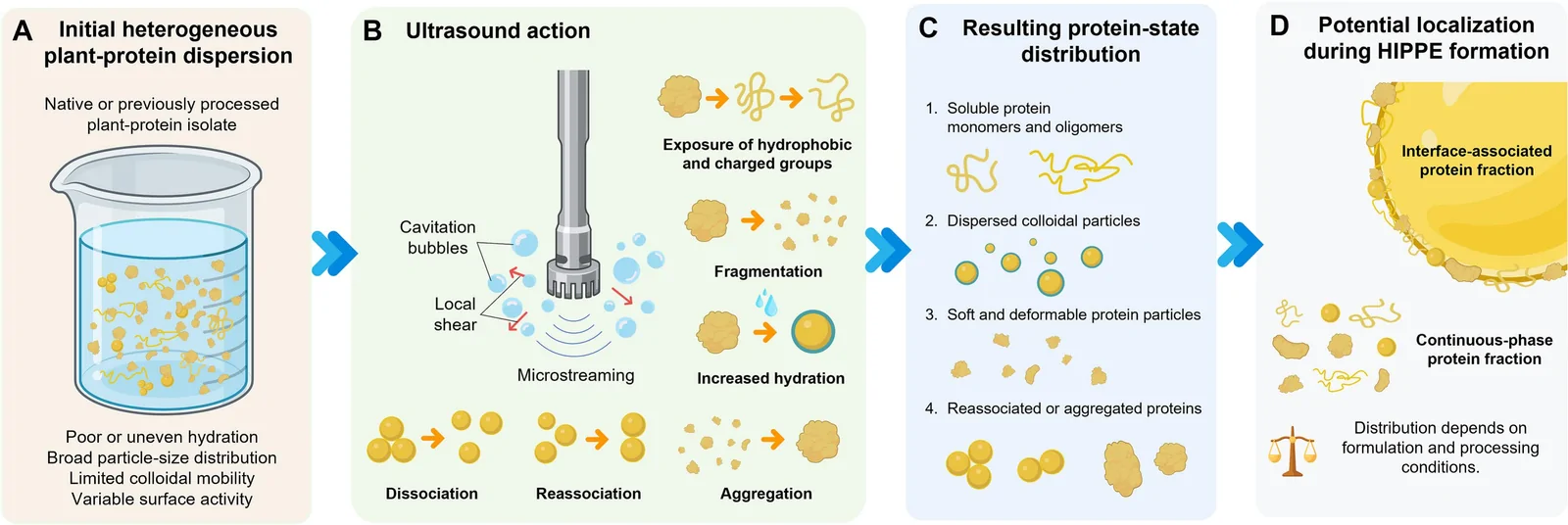
Ultrasound-induced redistribution of plant-protein states relevant to HIPPE stabilization. (A) An initially heterogeneous plant-protein dispersion contains poorly or unevenly hydrated aggregates, colloidal particles, and soluble proteins or oligomers. (B) Cavitation, local shear, and microstreaming promote changes in protein states such as fragmentation, dissociation, increased hydration, exposure of hydrophobic or charged groups, reassociation, and aggregation. (C) These competing effects redistribute the population among soluble monomers and oligomers, dispersed colloidal particles, soft deformable particles, and reassociated or aggregated proteins. (D) During HIPPE formation, these populations may partition between the oil–water interface and the continuous aqueous phase according to formulation and emulsion-processing conditions.

## 4 Protein distribution and function at the interface and in the continuous phase

### 4.1 Interfacial adsorption and protein layer formation

At the oil–water interface, stabilization begins when proteins or protein particles are transported to newly formed oil droplet surfaces. Smaller proteins and particles generally reach the interface more rapidly than larger aggregates, and direct ultrasonic emulsification can further enhance protein transport to the interface by promoting mixing and droplet breakup. As droplet size decreases, the total interfacial area increases, increasing the amount of protein required for surface coverage. If protein adsorption is too slow, incomplete surface coverage can allow droplets to recoalesce (J. Sun et al., 2025; T. Wang et al., 2022; L. Zhang et al., 2023).

Once proteins reach the oil–water interface, they need suitable affinity for both oil and aqueous phases to remain adsorbed and form a stable interfacial layer. This property is related to particle wettability, which is influenced by surface hydrophobicity and hydration. Moderate exposure of hydrophobic regions can strengthen interactions with the oil phase, while sufficient hydration helps maintain protein dispersion before adsorption. Contact angle is useful for comparing relative wettability among samples, but measurements made on dried protein pellets may not accurately reflect the behavior of hydrated protein particles at the oil–water interface (W. Chen et al., 2023; Ma et al., 2025; Nimaming et al., 2024; X. Sun et al., 2023; Y. Sun et al., 2022; W. Wang et al., 2022; Yu et al., 2025; R. Zhang et al., 2025). The importance of these surface properties is illustrated by soybean lipophilic protein treated at different ultrasound powers. At pH 2.0, samples were treated for 10 min at 0–450 W, and the most favorable interfacial properties were observed at 350 W. Under this condition, the adsorbed protein percentage reached approximately 53%, interfacial coverage became more uniform, and freeze–thaw stability improved (Y. Sun et al., 2022). Increasing the ultrasound power beyond this condition did not produce further improvement.

In addition to wettability, the amount of protein adsorbed at the interface is important for evaluating droplet surface coverage. The adsorption percentage should be considered together with the protein-to-oil ratio and the total interfacial area. At a low protein-to-oil ratio, even a high adsorption percentage may not provide enough protein for interfacial coverage. At a high protein-to-oil ratio, excess protein may form multilayers or accumulate as aggregates near the interface. In this regard, measuring the amount of adsorbed protein per unit interfacial area is useful for quantifying interfacial protein loading. Because accurate determination of this value requires estimation of the total interfacial area, the complete droplet-size distribution should also be measured, especially when a large number of small droplets are present.

The study of heat-treated faba bean and pea proteins illustrates why the amount of adsorbed protein should be interpreted together with interfacial structure. Concentrated Pickering emulsions were prepared using 4% (*w*/*v*) protein dispersions at an oil fraction of 0.70. After sonication at 20 kHz and 120 W for 5–20 min, a more uniform interfacial layer was observed even when the measured adsorbed fraction and interfacial protein concentration were relatively low (Lee et al., 2025). This result suggests that a relatively low amount of adsorbed protein can be associated with uniform interfacial coverage, highlighting the importance of protein distribution across the droplet surface.

The composition of the interfacial layer is another important factor in understanding the stabilization mechanism. In plant-protein HIPPEs, colloidal protein particles can provide particle-mediated stabilization, while soluble proteins can contribute to interfacial stabilization. The relative contribution of these protein forms depends on the structural state produced by ultrasound treatment and should be assessed directly where possible. For example, analysis of adsorbed and unadsorbed fractions in ultrasound-treated rice bran protein–EGCG systems showed that a portion of the treated protein remained unadsorbed, indicating that not all protein was incorporated into the interfacial layer (H. Li et al., 2025).

Once colloidal protein particles adsorb at the oil–water interface, they can undergo deformation and interact with neighboring particles. Moderate deformation can help fill gaps between adsorbed particles and improve the uniformity of interfacial coverage. When particle–particle attraction becomes too strong, particles can form clusters before or after adsorption, leaving some areas of the droplet surface insufficiently covered. The importance of controlling particle aggregation for interfacial coverage and emulsion stability is illustrated by studies involving high-power ultrasound treatment of chickpea protein (Cui et al., 2025) and ultrasound treatment of soybean lipophilic protein (Y. Sun et al., 2022).

These changes in the interfacial layer can be evaluated using interfacial rheology and interfacial tension measurements. Interfacial shear and dilatational rheology can show whether the adsorbed layer is weak and easily deformed or forms a stronger structure that resists compression and shear. Dynamic interfacial tension provides information on protein adsorption kinetics, but a rapid decrease in tension does not necessarily indicate the formation of a densely packed particle layer because soluble proteins can produce a similar response. A clearer assessment of interfacial structure requires surface pressure, compression–expansion behavior, and interfacial moduli to be considered together with interfacial imaging and the amount of protein adsorbed per unit area (Igartúa et al., 2024; C. Liu et al., 2025; Lv et al., 2025; N. Wang et al., 2023; Wen, Zhao, Jiang, & Sui, 2024; Wen, Zhao, Zhang, et al., 2024; Xie et al., 2027).

Interfacial mechanics can change with time and deformation history. Experimental reports should specify the interval between interface formation and testing and, where relevant, assess the interfacial layer's resistance to repeated deformation and its ability to recover. Compression, stress relaxation, and creep tests can help determine whether the interfacial layer remains functional under stresses associated with pumping, spreading, printing, or oral processing.

### 4.2 Unadsorbed proteins and continuous-phase structuring

Proteins that do not adsorb at the oil–water interface remain in the aqueous continuous phase. As the oil fraction increases, the aqueous-phase volume decreases, which can increase the concentration of unadsorbed proteins and particles, depending on protein loading and interfacial adsorption. Their contribution depends on their size and aggregation state. Dispersed particles can increase the viscosity of the continuous phase and reduce aqueous-phase drainage, while larger aggregates can bridge neighboring droplets. Protein–polysaccharide complexes can also retain water and contribute to network formation in the continuous phase.

These continuous-phase effects can become unfavorable when protein aggregation is excessive. Large flocs can make the emulsion structure more heterogeneous, promote local water release, and interfere with mixing. Excessively high viscosity in the continuous phase can also limit droplet breakup during emulsification. A continuous-phase network can support HIPPE stability, but its contribution should be considered together with the extent of protein coverage at the droplet interface.

The balance between interfacial and continuous-phase contributions becomes more complex when other stabilizing components are present. In ultrasound-treated pea protein–mung bean starch complexes, protein adsorption at the droplet surface occurred together with water immobilization and network formation in the continuous phase (S. Li et al., 2024). A similar pattern was reported for ultrasound- and pH-shifted pea protein combined with chitosan particles, where different components contributed to interfacial stabilization and continuous-phase structuring (J. Zhang, Zhao, Liu, et al., 2023). In these systems, changes in viscosity or network strength cannot be attributed exclusively to protein, because starch or chitosan can also contribute to the continuous-phase structure.

The distribution of protein between the interface and continuous phase depends on formulation. Aggregation before adsorption can leave the interface insufficiently covered, whereas extensive adsorption can reduce the amount of protein available to contribute to continuous-phase viscosity and drainage resistance. Because protein distribution and aggregation state can change during storage through adsorption, desorption, and reassociation, adsorbed and unadsorbed fractions should be characterized separately at relevant storage times (H. Li et al., 2025; Wen, Zhao, Zhang, et al., 2024; J. Sun et al., 2025; X. Sun et al., 2023; T. Wang et al., 2022).

Fig. 2 summarizes the complementary contributions of interfacial adsorption and continuous-phase structuring to HIPPE stability.

**Fig. 2:**
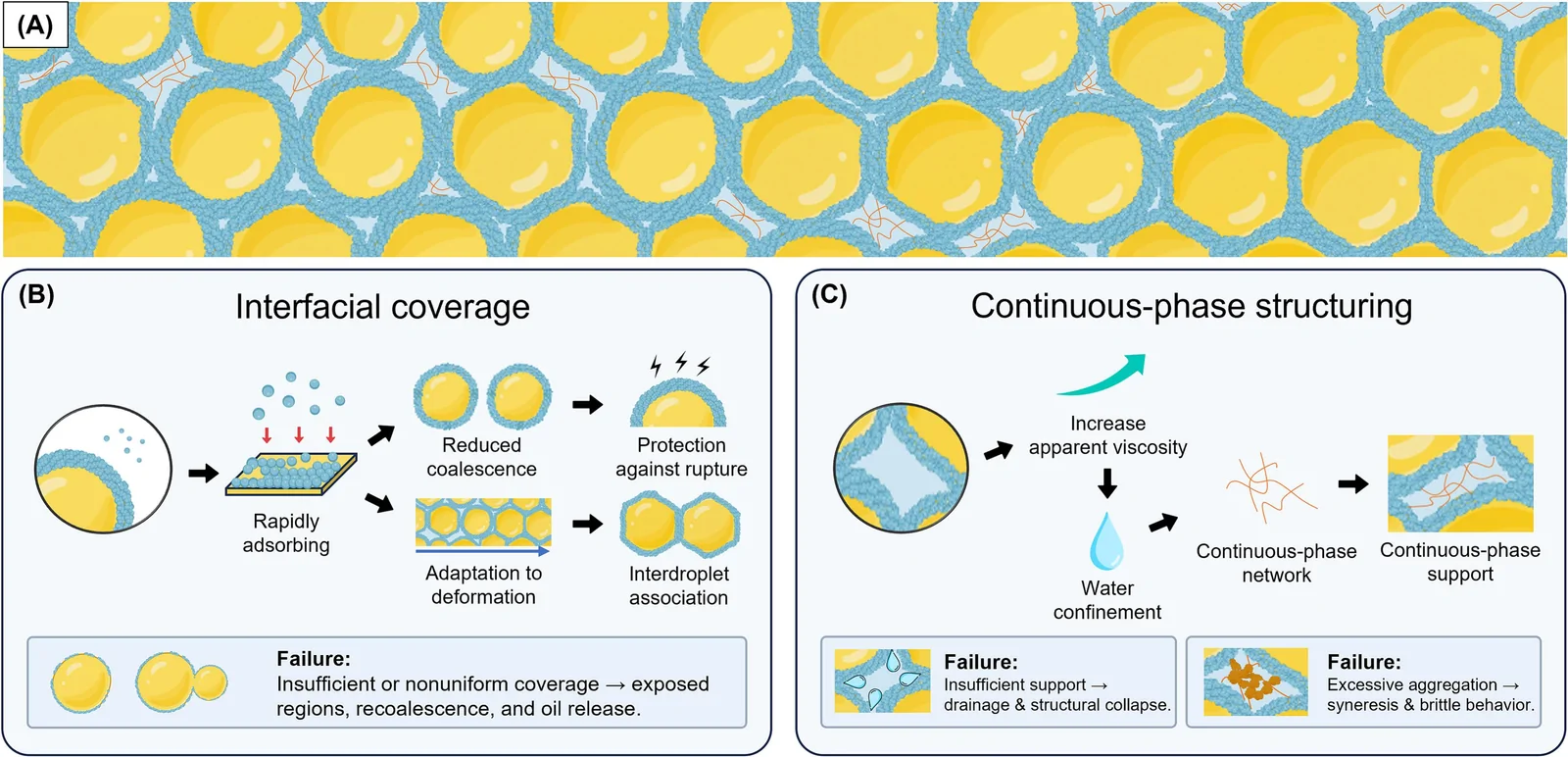
Coupled interfacial coverage and continuous-phase networking in plant-protein HIPPEs. (A) Closely packed droplets are protected by interfacial protein or particle layers and supported by proteins or aggregates in the restricted continuous aqueous phase. (B) Interfacial stabilization involves rapid adsorption, sufficient surface coverage, reduced coalescence, adaptation to deformation, and protection against rupture. Insufficient or nonuniform coverage leaves exposed regions and promotes recoalescence and oil release. (C) Continuous-phase networking increases apparent viscosity, confines water, and forms a load-bearing structure. Insufficient support promotes drainage and structural collapse, whereas excessive aggregation can cause syneresis and brittle behavior.

### 4.3 Effects of formulation variables on ultrasound performance

The outcome of ultrasound depends on protein concentration and oil fraction. At a fixed protein concentration, increasing the oil fraction reduces the volume of the aqueous phase and can increase the interfacial area that must be covered relative to the available protein. At a fixed oil fraction, the formation of smaller droplets increases the total interfacial area and requires more protein for adequate surface coverage. As a result, ultrasound conditions that stabilize one formulation may become insufficient when the oil fraction increases or smaller droplets are produced.

The effect of protein concentration on ultrasound-assisted high-internal-phase emulsion formation was demonstrated using pea protein. The ultrasound–pH-shift treatment was performed at 20 kHz, 500 W, and 10 min, while the concentration of modified protein varied from 1 to 5 wt% at an oil fraction of 0.75. Increasing the protein concentration progressively reduced droplet size and increased viscoelasticity, with 5 wt% producing the most stable and printable HIPPE (J. Zhang, Zhao, Li, et al., 2023). This result shows that protein concentration can affect emulsion formation and stability even under identical ultrasound conditions. Thus, ultrasound conditions should be evaluated together with the formulation used for HIPPE preparation.

## 5 Structure, rheology, and failure modes of plant-protein HIPPEs

HIPPE rheology reflects droplet packing, interfacial mechanics, interdroplet interactions, and continuous-phase structuring, all of which can be altered directly or indirectly by ultrasound.

### 5.1 Droplet packing and rheological behavior

Droplet-size distribution affects droplet packing in HIPPEs. Small droplets can fill the spaces between larger droplets and increase packing efficiency. By comparison, a narrow droplet-size distribution can promote more uniform droplet contacts and deformation at high oil fractions. Because ultrasound can change both the average droplet size and the width of the distribution, HIPPE structure should be evaluated using the full droplet-size distribution together with microscopy (Aryanti et al., 2025b; He & Lu, 2024; Ji & Luo, 2023).

These differences in droplet packing are reflected in the rheological behavior of HIPPEs. Many plant-protein HIPPEs show shear-thinning behavior and exhibit G′ > G″ under small-amplitude oscillatory shear, indicating a predominantly elastic response within the linear viscoelastic region. However, a higher G′ can result from several structural contributions, including close droplet packing, attractive interactions between droplets, protein bridging, interactions among particles in the continuous phase, and polysaccharide thickening (X. Chen et al., 2023; Igartúa et al., 2024; C. Liu et al., 2025; X.-Y. Liu et al., 2024; Lv et al., 2025; Rawal et al., 2025; Xie et al., 2027; L. Zhu et al., 2025). Thus, G′ should not be interpreted as direct evidence of stronger protein adsorption at the oil–water interface.

Oil fraction can influence this rheological response because increasing the dispersed-phase volume increases droplet crowding. For example, modified soy-protein emulsions showed increased rheological strength as the fraction of flaxseed-derived diglyceride increased, partly because the droplets became more closely packed (Badar, Wang, Sun, et al., 2024). When ultrasound treatments are compared, the oil fraction should be held constant, or its influence should be assessed together with droplet size and protein-to-oil ratio.

For practical applications, HIPPEs must resist phase separation at rest while deforming and recovering during pumping, spreading, swallowing, or extrusion. Yield stress and thixotropic recovery provide practical measures of resistance to flow and structural recovery after shear, respectively.

Because these rheological properties are sensitive to the testing conditions, reliable comparisons require consistent measurement procedures. Rest time before measurement, pre-shear conditions, the strain amplitude selected within the linear viscoelastic region, methods used to minimize wall slip, the method used to determine yield stress, and the shear and recovery conditions used for thixotropic testing should be clearly reported. Without this information, variation arising from the testing protocol may be misinterpreted as an effect of formulation or ultrasound treatment.

### 5.2 Failure during storage and processing

Failure of HIPPEs can arise from instability at the oil–water interface, in the continuous phase, or within the droplet network. Incomplete interfacial coverage can promote coalescence, whereas insufficient continuous-phase support can lead to drainage. Excessive bridging can produce heterogeneous flocculation and syneresis, while an overly rigid interfacial or continuous-phase network may fracture under large deformation.

Drainage and syneresis can occur when the aqueous phase is not sufficiently retained between closely packed droplets. Polysaccharides and unadsorbed protein particles can increase water retention and limit aqueous-phase separation. However, excessive structuring of the continuous phase can make emulsification more difficult and may cause the network to fracture under shear (Aryanti et al., 2025a; Diao et al., 2024; R. Li et al., 2025; J. Zhang, Zhao, Liu, et al., 2023).

These failure modes may become more apparent when HIPPEs are exposed to environmental or mechanical stresses. Heating, changes in pH and salt concentration, and freeze–thaw cycling can alter protein interactions, interfacial structure, and droplet stability, while shear during processing can disrupt the network and affect its ability to recover after deformation. Stability tests should reflect the stresses that HIPPEs are likely to experience during processing, storage, and consumption (Qin, Luo, Peng, et al., 2018; J. Sun et al., 2025; Yu et al., 2025).

In addition to the physical changes caused by these stresses, chemical stability should be evaluated separately. In oil-containing systems, direct ultrasonic emulsification can cause local heating and radical formation, while metal particles released from the ultrasonic probe may further promote lipid oxidation. Thus, an emulsion may show improved physical stability while undergoing increased oxidative deterioration. For systems containing unsaturated oils or receiving prolonged ultrasound treatment, primary and secondary lipid oxidation products should be monitored (Akhtar & Masoodi, 2022; W. Chen et al., 2023; X. Chen et al., 2023; Diao et al., 2024; N. Wang et al., 2023).

To evaluate physical stability under deformation, large-deformation and recovery tests can help determine whether structural breakdown is reversible or permanent. Coalescence, oil leakage, aqueous-phase separation, syneresis, phase inversion, and a permanent decrease in modulus can indicate irreversible structural failure. Microscopy before and after stress application can further distinguish droplet flocculation, deformation, and coalescence.

Overall, a suitable HIPPE formulation should maintain sufficient interfacial protection, limit drainage, and recover after the stresses encountered during intended use. The next section examines whether reported measurements directly support the proposed stabilization mechanisms.

## 6 Reassessment of stabilization mechanisms and formulation requirements

Commonly reported outcomes, such as smaller droplets, higher apparent viscosity, increased G′, and slower phase separation, indicate changes in emulsion structure or stability but do not identify the underlying stabilization mechanism. Mechanistic assessment requires measurements that localize stabilizing components and distinguish interfacial from continuous-phase contributions.

### 6.1 Evidence for evaluating stabilization mechanisms

Reported systems can be classified as interfacial-dominant, continuous-phase-dominant, coupled, or unresolved according to the available evidence. An interfacial-dominant interpretation requires direct evidence that proteins or protein particles are present at the oil–water interface and contribute to its stabilization. Useful measurements include the amount of adsorbed protein normalized by the total interfacial area, interfacial imaging that identifies the stabilizing component, desorption measurements, and interfacial shear or dilatational rheology. Adsorption percentage, dynamic interfacial tension, and droplet size can support this interpretation but are insufficient on their own because these measurements can also be influenced by soluble proteins, multilayer adsorption, or droplet breakup (Lee et al., 2025; H. Li et al., 2025; Y. Sun et al., 2022; Wen, Zhao, Zhang, et al., 2024).

By contrast, a continuous-phase-dominant interpretation requires direct evidence that proteins or particles remaining in the continuous phase contribute to emulsion stability. Useful measurements include the concentration and particle size of unadsorbed proteins, continuous-phase rheology, matched oil-free controls, water-mobility measurements, and imaging of the aqueous phase. Bulk G′ or water-holding capacity is not sufficient to confirm the formation of a continuous-phase network because similar changes can result from droplet crowding.

When both regions contribute to stabilization, a coupled mechanism requires independent evidence of contributions from both the interface and the continuous phase. Such evidence may include direct characterization of the interfacial layer together with evidence of continuous-phase network formation, restricted water mobility, or the contribution of a separately characterized co-stabilizer (Lee et al., 2025; H. Li et al., 2025; S. Li et al., 2024; J. Zhang, Zhao, Liu, et al., 2023). If one region has not been directly examined, its contribution should be considered unresolved and not inferred from bulk measurements.

This unresolved category is important because gelation does not necessarily indicate improved Pickering stabilization. For example, a concentrated soy-protein emulsion treated with short probe sonication showed droplet breakup, close packing, bridging, and noncovalent interactions (Xie et al., 2027). However, the system contained only 60% oil, below the composition normally associated with HIPPEs. Thus, this study provides useful information on ultrasound-induced structural changes but does not provide direct evidence for particle restructuring in HIPPEs.

### 6.2 Measurements and controls needed for formulation design

To obtain the evidence needed for this evaluation, experimental design should specify both the material exposed to ultrasound and the formulation conditions during treatment. Treatment sequence, protein concentration, oil fraction, pH, sample volume, temperature history, equilibration time, and the order of co-stabilizer addition should be clearly reported because these variables can influence the effects of ultrasound on the system.

Accordingly, appropriate controls are needed to distinguish protein modification from droplet breakup. Pre-sonication of proteins followed by non-ultrasonic emulsification helps isolate the contribution of protein modification, whereas direct ultrasonic emulsification includes both protein modification and droplet breakup. Temperature-matched controls are needed when ultrasound causes appreciable heating. For hybrid treatments, ultrasound-only, secondary-treatment-only, and combined treatments should be compared (Adamou et al., 2024; Ampofo & Ngadi, 2022; X. Zhu et al., 2024).

Formulation comparisons should control droplet size and continuous-phase viscosity where feasible because either can alter emulsion stability independently of changes at the interface. Protein-to-oil ratio should be reported together with the full droplet-size distribution. Estimating the total interfacial area allows the amount of adsorbed protein to be expressed per unit area, providing a more comparable basis for evaluating treatments across formulations.

After accounting for formulation differences, large-deformation tests can be used to evaluate how HIPPEs fail and recover during pumping, spreading, swallowing, or printing. Yield stress, stress relaxation, creep, thixotropic recovery, and fracture testing provide complementary information on resistance to deformation and structural recovery. Interfacial compression–expansion measurements can further determine how the adsorbed layer responds to deformation (Wen, Zhao, Jiang, et al., 2024; Wen, Zhao, Zhang, et al., 2024).

For practical applications, measurement selection should be guided by the intended use of the HIPPE. Printable HIPPEs require controlled extrusion and rapid structural recovery, whereas spreads require moderate yield stress and limited oil release. Swallowing-oriented foods should remain cohesive without becoming excessively adhesive, while delivery systems need to maintain stability during storage and permit oil-phase release during gastrointestinal digestion (X. Chen et al., 2023; Han et al., 2024; R. Li et al., 2025; Rawal et al., 2025; Z. Sun et al., 2025; Wei et al., 2025).

Support for a coupled stabilization mechanism requires evidence from both the interface and the continuous phase, together with product-relevant stress testing when functional performance is claimed. Table 2 summarizes the analytical methods used at different scales, the information provided by each method, and their main limitations.

**Table 2:** Multiscale analytical requirements for distinguishing particle-mediated interfacial stabilization from molecular protein adsorption and continuous-phase structuring.

Scale	Property or question	Recommended methods	What the measurement supports	Important limitation	References
Acoustic process	How much energy reaches the sample?	Calorimetry; specific energy input; temperature-time recording; cavitation dosimetry or hydrophone where available.	Comparison of actual energy delivery and separation of acoustic from thermal effects.	Nominal power, amplitude percentage, and final temperature are not transferable descriptors.	Adamou et al., 2024; Ampofo & Ngadi, 2022; X. Zhu et al., 2024
Bulk particle population	Are persistent colloidal particles present?	DLS or laser diffraction with full distributions; microscopy; nanoparticle tracking; analytical centrifugation; filtration/fractionation.	Particle size, heterogeneity, soluble/particulate fractions, and persistence after equilibration.	Intensity-weighted size is biased toward large scatterers; “soluble” depends on operational separation conditions.	Cui et al., 2025; Lee et al., 2025; Y. Wang et al., 2022; Zhao et al., 2022
Protein conformation	How has ultrasound changed molecular exposure and association?	Intrinsic fluorescence; surface hydrophobicity; FTIR/CD; free sulfhydryl/disulfide assays; solubility.	Partial unfolding, exposure of hydrophobic/reactive groups, and changes in intermolecular bonding.	Measurements may be dominated by soluble fractions and do not directly demonstrate interfacial function.	Das et al., 2022; Gharibzahedi & Smith, 2020; Jadhav et al., 2024; Y. Wang et al., 2022
Colloidal interaction/wettability	Can particles remain dispersed and prefer the oil–water interface?	ζ-Potential; contact angle; interfacial partitioning; hydration and sedimentation tests.	Electrostatic stability and relative oil/water affinity.	Contact angles on dried compacts may not represent hydrated soft particles; ζ-potential is not surface charge density.	W. Chen et al., 2023; Ma et al., 2025; Nimaming et al., 2024; Y. Sun et al., 2022
Adsorption kinetics	How rapidly does stabilizer occupy newly formed interface?	Dynamic interfacial tension or surface pressure; time-resolved adsorption.	Transport and adsorption kinetics during interface generation.	Rapid tension reduction may be caused mainly by soluble proteins and does not prove particle jamming.	Sui et al., 2017; T. Wang et al., 2022; L. Zhang et al., 2023
Interfacial localization/composition	Are intact particles actually present at the interface?	Confocal microscopy with separate labels; cryo-EM; AFM of transferred interfaces; analysis of isolated cream layer; adsorbed-protein quantification.	Direct localization, surface coverage, and interfacial composition.	Phase separation can disturb weakly adsorbed material and adsorbed mass cannot distinguish molecules from particles.	H. Li et al., 2025; Wen, Zhao, Zhang, et al., 2024; L. Zhang et al., 2023
Interfacial mechanics	Does the layer resist compression, dilation, and shear?	Interfacial shear and dilatational rheology; compression/expansion cycles; desorption or displacement tests.	Elasticity, cohesion, rearrangement, jamming, and desorption resistance.	Strong mechanics may arise from mixed films or cross-linked layers, not necessarily classical rigid-particle Pickering layers.	Sui et al., 2017; T. Wang et al., 2022; H. Li et al., 2025
Droplet and HIPPE microstructure	How are droplets packed and deformed?	Optical/confocal microscopy; droplet-size distribution; cryo-imaging; water-film and phase-release observations.	Coverage uniformity, polyhedral deformation, crowding, flocculation, and drainage.	Small droplet size does not identify the stabilization mechanism.	Lee et al., 2025; Cui et al., 2025; Qin, Luo, & Peng, 2018; Qin, Luo, Peng, Lu, et al., 2018
Bulk network/rheology	What generates the self-supporting structure?	Strain/frequency sweeps; yield stress; flow curves; thixotropic recovery; creep; LAOS; tribology where relevant.	Elasticity, recoverability, bridging, continuous-phase network formation, and application flow.	High G′ may originate from crowding, polysaccharide thickening, bridging, or interfacial strength.	Badar, Wang, Chen, et al., 2024; Lee et al., 2025; L. Zhu et al., 2025
Stability/application	Does the structure survive realistic use?	Storage, heating/cooling, freeze–thaw, centrifugation, oil/water release, oxidation, digestion, printing, texture, and product tests.	Functional validity under intended processing and consumption conditions.	Short-term visual stability in a model emulsion is insufficient for application claims.	Q. Yang et al., 2025; Y. Wang et al., 2026; L. Zhu et al., 2025

## 7 Scale-up considerations

Laboratory probe sonicators are useful for screening, but their acoustic field, mixing, heat transfer, and residence-time distribution differ substantially from those of food-scale processing systems. During scale-up, operating conditions should be selected to reproduce the measured acoustic exposure and the required protein or emulsion properties. Equivalent processing conditions are unlikely to be achieved by directly scaling amplitude, nominal power, or treatment time (Adamou et al., 2024; Ampofo & Ngadi, 2022; X. Zhu et al., 2024).

### 7.1 Acoustic energy reporting and reactor scale-up

Quantitative comparisons of ultrasound treatments require the measurement and reporting of acoustic exposure whenever possible. Generator power and amplitude percentage are equipment-specific settings and cannot be directly compared among systems. Relevant parameters include frequency, nominal and calorimetrically measured power where available, specific energy, probe diameter, immersion depth, vessel geometry, sample volume, pulse mode, duty cycle, and flow or stirring conditions. These parameters should be reported together with the complete temperature history, protein concentration, and oil fraction (Adamou et al., 2024; Ampofo & Ngadi, 2022; X. Zhu et al., 2024).

Temperature monitoring throughout ultrasound treatment is important because heating can independently affect protein unfolding, aggregation, and interfacial behavior. An ice bath can control the bulk temperature but cannot eliminate local heating and intense shear near the probe. Temperature-matched controls help separate mechanical effects from thermal effects. Ultrasound treatments that intentionally include heating are more appropriately described as thermosonication treatments.

Spatial variation in acoustic exposure is another important consideration during scale-up. Batch probe systems generate a non-uniform acoustic field, with material near the probe tip receiving greater exposure than material near the vessel walls. This non-uniformity can become more pronounced as protein concentration and HIPPE viscosity increase because high viscosity can restrict liquid circulation and change cavitation behavior. The extent of this spatial variation depends on probe diameter, immersion depth, vessel dimensions, mixing conditions, and treatment volume, which together determine the acoustic exposure experienced by the sample (Adamou et al., 2024; X. Zhu et al., 2024).

Continuous-flow and recirculating reactors can address some of these scale-up limitations by increasing throughput and improving heat removal, although they introduce additional challenges related to residence-time distribution and uniform acoustic exposure. Flow rate, number of passes, hold-up volume, and sampling position need to be clearly reported. For final HIPPE production, staged processing may offer a practical approach. A low- or moderate-viscosity protein dispersion can first be sonicated before oil is added to achieve the target oil fraction, avoiding the greater acoustic attenuation associated with direct treatment of highly concentrated systems (Adamou et al., 2024; X. Zhu et al., 2024).

Beyond reactor configuration, food-scale implementation also requires attention to fouling, cleanability, sanitation, and equipment wear. Protein or hydrocolloid deposition on probe or flow-cell surfaces can alter energy transfer and increase cleaning requirements, while immersed sonotrodes may undergo pitting or erosion during prolonged operation (Adamou et al., 2024; X. Zhu et al., 2024). Hygienic design should facilitate clean-in-place operation or easy disassembly, minimize dead zones, use food-compatible wetted materials, and establish inspection and replacement criteria for sonotrodes. For direct-contact ultrasound applied to oxidation-sensitive foods, metal release associated with sonotrode wear should be monitored.

Scale-up is further affected by raw-material variability. Commercial protein isolates can differ in denaturation history, solubility, aggregate size, ash content, carbohydrate content, and residual lipids. These lot-to-lot differences can alter acoustic attenuation and the ultrasound conditions required to achieve the target particle size and properties. At-line measurements of particle size, turbidity, viscosity, temperature, or acoustic response can provide more reliable process control than relying on a fixed treatment time.

For industrial evaluation, ultrasound needs to be benchmarked against conventional methods such as rotor–stator and high-pressure homogenization using comparable processing criteria. Equal treatment time does not provide a meaningful basis for comparison because these methods can produce different droplet-size distributions and thermal histories. Comparisons can be based on product quality at similar droplet-size distributions or stabilizer loadings, together with specific energy consumption, throughput, cooling requirements, product yield, and maintenance requirements (Adamou et al., 2024; Ampofo & Ngadi, 2022; X. Zhu et al., 2024).

Beyond process performance, economic feasibility depends on both capital and operating costs. Relevant factors include investment in reactors and transducers, throughput, electricity consumption, cooling demand, cleaning and sanitation time, probe or sonotrode replacement, pumping requirements, product losses, and batch-to-batch variability. An increase in G′ by itself provides limited justification for higher processing costs. The added cost is more readily justified when ultrasound reduces stabilizer use, replaces another unit operation, improves product yield, or provides a functional benefit that cannot be achieved by conventional emulsification.

Fig. 3 integrates the evidence requirements, product validation, and scale-up considerations into a practical workflow. The workflow begins with quantification of acoustic and formulation inputs, followed by characterization of particle, interfacial, and continuous-phase properties and evidence-based interpretation of the stabilization mechanism. Product-specific validation is then combined with a scale-up process that progresses from batch screening to recirculating or flow-through processing and pilot-scale evaluation.

**Fig. 3:**
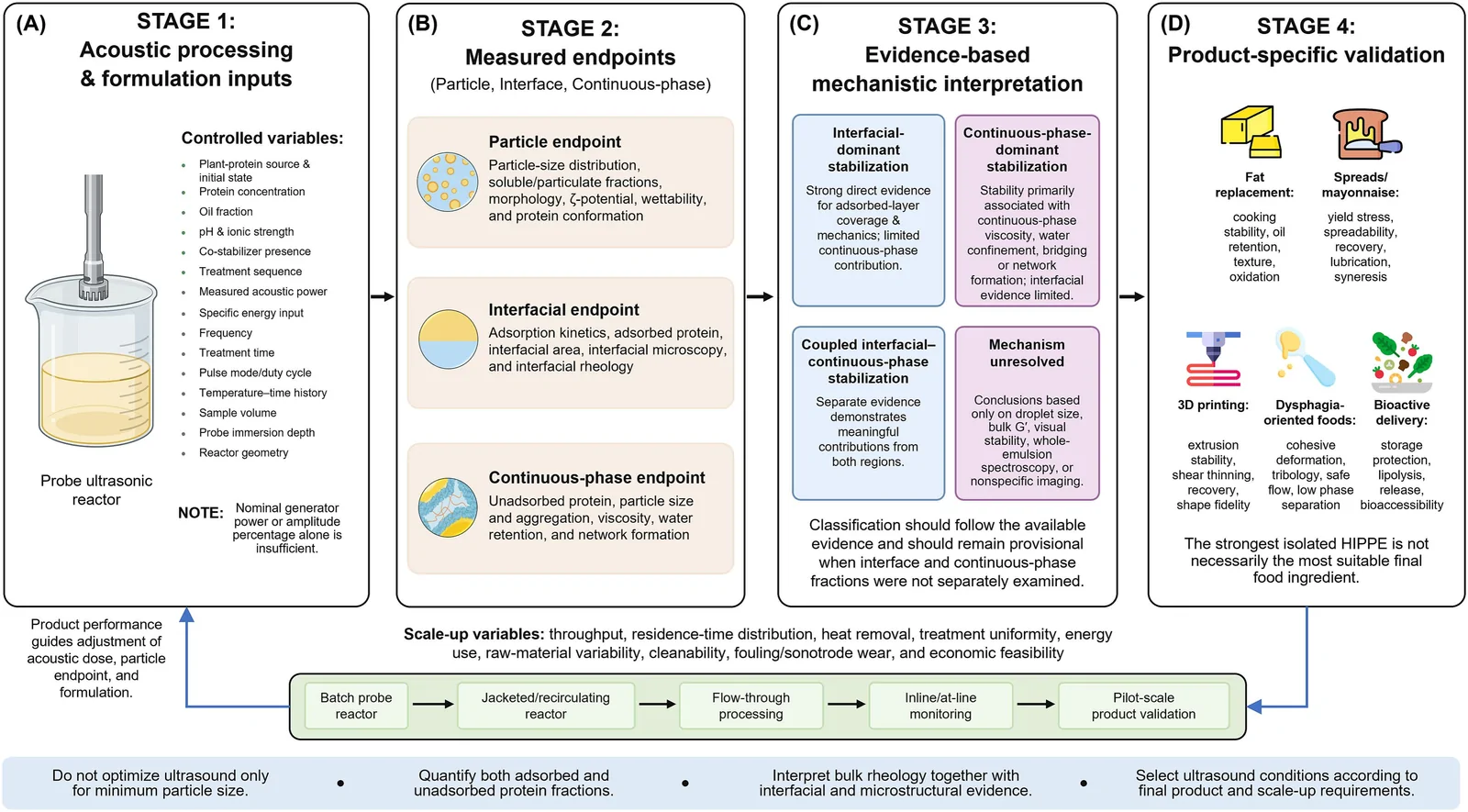
Practical evaluation and design of ultrasound-engineered plant-protein HIPPEs. The workflow links acoustic-processing and formulation inputs to measured particle, interfacial, and continuous-phase endpoints, mechanistic interpretation, product validation, and scale-up. (A) Stage 1 emphasizes complete reporting of formulation variables and acoustic conditions, including measured power, specific energy, frequency, treatment time, pulse mode, temperature history, sample volume, probe immersion depth, and reactor geometry; nominal power or amplitude percentage is insufficient. (B) Stage 2 separately characterizes particle states, interfacial adsorption and mechanics, and the unadsorbed protein fraction and continuous-phase network. (C) Stage 3 classifies stabilization as interfacial-dominant, continuous-phase-dominant, coupled, or unresolved according to the available evidence. (D) Stage 4 validates performance in representative food applications.

## 8 Food applications and product validation

At the product level, a HIPPE that is stable in a model system may behave differently when incorporated into a food matrix. Salts, acids, starches, hydrocolloids, endogenous proteins, and heating can alter both the interfacial layer and the continuous phase. Product validation should determine whether the droplets remain intact, whether oil or water is released, and whether the HIPPE maintains its structure within the surrounding food matrix.

For fat replacement, an ultrasound-modified almond-protein HIPPE was incorporated into pork sausage (Q. Yang et al., 2025). In this application, the key criterion was whether the HIPPE retained oil during mixing and cooking. Cooking yield, texture, microstructure, oxidative stability, and sensory quality provided more meaningful evidence of product performance than G′ measured for the isolated HIPPE.

Spreads and mayonnaise-like foods require application-specific evaluation because they need stability at rest together with smooth shear-thinning behavior and structural recovery during use. For example, a study of ultrasound-assisted walnut-oil Pickering emulsions applied to mayonnaise showed the importance of evaluating yield stress, spreadability, oil and water separation, lubrication, and oxidative stability (Akhtar & Masoodi, 2022). These product-level properties provide a more relevant basis for evaluation than small-strain elasticity.

Three-dimensional printing also requires application-specific rheological evaluation. Printable HIPPEs require appropriate shear-thinning behavior during extrusion and rapid structural recovery after deposition. Extrusion pressure, filament continuity, dimensional accuracy, and post-printing recovery provide direct criteria for evaluating printability. In contrast, a high modulus without sufficient recovery can lead to nozzle blockage or discontinuous extrusion (J. Song et al., 2023; Wu et al., 2024).

For swallowing-oriented foods, HIPPE formulations require cohesive deformation, predictable flow, low phase separation, and limited adhesiveness. Ultrasound-modified soy protein–hyaluronic acid systems have been used to prepare HIPPE gels for dysphagia-oriented foods and 3D printing (Huang et al., 2024; Y. Wang et al., 2026). These applications show that maximum gel strength is not the primary target; flow behavior, structural recovery, and ease of swallowing provide more relevant performance criteria.

For delivery applications, HIPPEs need to balance storage protection with digestive accessibility. Pea protein–mung bean starch and soy protein–β-glucan–catechin particle systems have been used to deliver β-carotene or curcumin (X. Chen et al., 2023; S. Li et al., 2024). In these systems, encapsulation retention, lipid digestion, and transfer of bioactives into mixed micelles need to be evaluated separately because an excessively rigid interfacial or continuous-phase network may improve retention during storage but limit release and bioaccessibility during digestion.

Collectively, these application examples show that the selection of ultrasound treatment route and stabilization strategy should reflect the intended use. Representative plant-protein systems, ultrasound processing routes, emulsion applications, and their main mechanistic interpretations are summarized in Table 3.

**Table 3:** Representative studies linking ultrasound-induced plant-protein restructuring to Pickering emulsion and HIPPE stabilization.

Protein/stabilizer system	Ultrasound or hybrid route	Emulsion type/application	Reported structural or functional outcome	Mechanistic point highlighted in this review	References
Quinoa protein nanoparticles	Protein pre-sonication and ultrasound-induced assembly	Pickering emulsions; HIPPEs	Changed nanoparticle assembly, interfacial adsorption/arrangement, rheology, and stability.	Demonstrates that ultrasound can generate reassembled, deformable particles that act at both interfacial and bulk levels.	Qin, Luo, & Peng, 2018; Qin, Luo, Peng, Lu, et al., 2018; X. Zhang et al., 2021
Chickpea protein nanoparticles	Power-dependent ultrasound fabrication	Pickering emulsion	Intermediate power reduced size and improved stabilization; highest power promoted particle growth/reaggregation.	Direct evidence for a non-monotonic response to ultrasound power.	Cui et al., 2025
Heat-treated faba bean and pea proteins	Sequential heating followed by different ultrasonication times	Concentrated Pickering emulsion (oil fraction 0.70)	Sonication time altered colloidal state, interfacial adsorption, and emulsion stability.	Optimal stability did not simply coincide with maximum adsorbed protein; layer uniformity and particle state were decisive.	Lee et al., 2025
Pea protein isolate	High-intensity ultrasound plus pH shifting	Emulsions; HIPPE with chitosan particles	Increased solubility and emulsification; generated strong concentrated emulsion structures.	Higher solubility may represent particles, oligomers, molecules, or mixtures and cannot prove Pickering behavior.	J. Zhang et al., 2022; J. Zhang, Zhao, Li, et al., 2023; J. Zhang, Zhao, Liu, et al., 2023
Oxidized soybean protein aggregates	Ultrasound treatment of pre-existing aggregates	O/W emulsion	Appropriate treatment depolymerized aggregates and improved interfacial properties; high power caused renewed association.	Ultrasound modifies but does not erase the protein's thermal or oxidative history.	Y. Wang et al., 2022
Ultrasound-modified almond protein isolate	Protein particle modification followed by HIPPE formation	Fat substitute in pork sausage	Intermediate treatment produced favorable HIPPE and sausage cooking, texture, and oxidative outcomes.	The optimal isolated HIPPE must still be validated within the final food matrix.	Q. Yang et al., 2025

Across these applications, validation should reproduce the environmental and mechanical stresses encountered during processing, storage, and use. Physical and chemical stability should be evaluated separately because a HIPPE that remains physically stable can still undergo lipid or protein oxidation and associated quality deterioration (Akhtar & Masoodi, 2022; W. Chen et al., 2023; Diao et al., 2024; Qin, Luo, Peng, Lu, & Zou, 2018; J. Sun et al., 2025; N. Wang et al., 2023).

Ultimately, ultrasound conditions should be selected based on product performance at the intended protein-to-oil ratio and oil fraction. The selected conditions should produce consistent results across raw-material lots and reactor scales while avoiding unnecessary energy input. Table 4 summarizes the corresponding product-specific performance targets, validation methods, and major failure risks.

**Table 4:** Application-specific design targets for ultrasound-engineered plant-protein Pickering emulsions and HIPPEs.

Target application	Required emulsion/HIPPE behavior	Particle/interface design priority	Recommended validation	Main failure risk	References
Fat replacement in meat products	Thermal stability, oil retention, compatibility with protein gelation, and acceptable texture.	Cohesive particle layer with a deformable but stable bulk network; avoid excessive rigidity.	Cooking loss, oil release, texture profile, microstructure, oxidation, and sensory evaluation in the meat matrix.	Discrete brittle inclusions, weak integration, oil leakage, or excessive hardness.	Q. Yang et al., 2025
Spreads, mayonnaise-like foods, and dressings	Yield stress at rest, smooth shear thinning, rapid recovery, and low syneresis.	Balanced droplet jamming and lubrication; uniform particles with limited bridging.	Flow curve, yield stress, thixotropy, tribology, spreadability, phase separation, and sensory smoothness.	Graininess, brittle fracture, poor spreadability, or water/oil separation.	Akhtar & Masoodi, 2022
3D-printed foods	Strong shear thinning through the nozzle and rapid post-extrusion recovery with dimensional fidelity.	Recoverable droplet/particle network with controlled yield stress and minimal phase release.	Nozzle pressure, extrusion continuity, filament width, shape fidelity, layer support, recovery, and post-print stability.	Nozzle blockage, strand collapse, over-rigid extrusion, or delayed oil/water release.	J. Song et al., 2023; Wu et al., 2024
Dysphagia-oriented formulations	Cohesive deformation, low separation, predictable flow, and safe swallowing texture.	Soft hydrated interfacial and bulk networks rather than maximum modulus.	Large-deformation rheology, cohesiveness, tribology, standardized swallowing tests, and storage stability.	Fragmentation in the mouth, residue, syneresis, or unsafe flow behavior.	Y. Wang et al., 2026
Bioactive delivery matrices	Protection during storage and controlled release/digestion with acceptable food texture.	Interfacial barrier and network should balance protection against accessibility and release.	Oxidative stability, encapsulation efficiency, digestion/lipolysis, bioaccessibility, release kinetics, and safety.	Overprotection that limits release, premature leakage, or oxidation promoted by large interfacial area.	X. Chen et al., 2023; S. Li et al., 2024; Han et al., 2024
Industrial scale-up	Reproducible acoustic dose, controlled temperature, uniform residence time, and acceptable energy use.	Mechanistically defined particle endpoint rather than fixed nominal settings.	Actual power, residence-time distribution, inline turbidity/size, temperature history, lot variability, and comparison with conventional homogenization.	Local overprocessing, untreated zones, probe erosion, inconsistent lots, or unfavorable energy/cooling demand.	Adamou et al., 2024; X. Zhu et al., 2024

## 9 Conclusions

High-intensity ultrasound does not universally improve the stabilization of plant-protein HIPPEs. Its effects depend on the ultrasound treatment route, protein source and structural state, treatment intensity, and formulation conditions. Pre-sonication allows protein modification to be separated from droplet formation, direct ultrasonic emulsification combines protein restructuring with droplet breakup and interfacial adsorption, and post-emulsification ultrasound acts on preformed droplets and established interfacial structures. The effectiveness of post-emulsification ultrasound also depends on the initial emulsion structure and the availability of protein in the continuous phase. Moderate treatment can improve protein particle properties and emulsion stability, whereas excessive treatment may promote reaggregation or reduce the desired functionality.

Protein concentration, protein-to-oil ratio, oil fraction, and droplet size further determine how ultrasound-induced changes affect HIPPE formation and stability. A clear understanding of the stabilization mechanism requires direct characterization of both the oil–water interface and the continuous phase. The amount of adsorbed protein per unit interfacial area, interfacial imaging or rheology, characterization of unadsorbed proteins, and route-specific controls provide more direct evidence of the stabilization mechanism, whereas droplet size, adsorption percentage, or bulk G′ can reflect several structural contributions.

For food applications, ultrasound conditions should be evaluated under product-specific processing, storage, and use conditions. Quantitative reporting of acoustic exposure, reactor configuration, temperature history, formulation, and treatment sequence is necessary for reproducibility and scale-up. Industrial translation further depends on heat removal, residence-time distribution, fouling, cleanability, equipment wear, throughput, and processing cost. Future studies should establish relationships among ultrasound conditions, protein structural changes, interfacial behavior, and final product performance, while validating the resulting HIPPEs in food systems under realistic processing and storage conditions.

## CRediT authorship contribution statement

**Uyory Choe:** Writing – review & editing, Writing – original draft, Visualization, Methodology, Investigation, Funding acquisition, Conceptualization.

## Funding

This work was supported by the 10.13039/501100003725National Research Foundation of Korea (NRF) grant funded by the Korean government (MSIT) (Grant No. RS-2026-25485983).

## Declaration of competing interest

The author declares that there are no known competing financial interests or personal relationships that could have appeared to influence the work reported in this paper.

## Data Availability

No new data were created or analyzed in this review. Data sharing is not applicable.
